# Extensive Characterization of Oxide-Coated Colloidal Gold Nanoparticles Synthesized by Laser Ablation in Liquid

**DOI:** 10.3390/ma9090775

**Published:** 2016-09-14

**Authors:** Romuald Intartaglia, Marina Rodio, Mohamed Abdellatif, Mirko Prato, Marco Salerno

**Affiliations:** 1 Nanophysics Department, Istituto Italiano di Tecnologia, Genoa I-16163, Italy; romuald.intartaglia@iit.it (R.I.); marina.rodio@iit.it (M.R.); 2 Nanostructures Department, Istituto Italiano di Tecnologia, Genoa I-16163, Italy; mohamed.abdellatif@iit.it; 3 Nanochemistry Department, Istituto Italiano di Tecnologia, Genoa I-16163, Italy; mirko.prato@iit.it

**Keywords:** gold nanoparticles, colloids, scanning Kelvin probe microscopy, surface oxide

## Abstract

Colloidal gold nanoparticles are a widespread nanomaterial with many potential applications, but their aggregation in suspension is a critical issue which is usually prevented by organic surfactants. This solution has some drawbacks, such as material contamination and modifications of its functional properties. The gold nanoparticles presented in this work have been synthesized by ultra-fast laser ablation in liquid, which addresses the above issues by overcoating the metal nanoparticles with an oxide layer. The main focus of the work is in the characterization of the oxidized gold nanoparticles, which were made first in solution by means of dynamic light scattering and optical spectroscopy, and then in dried form by transmission electron microscopy, X-ray diffraction, X-ray photoelectron spectroscopy, and finally by surface potential measurements with atomic force microscopy. The light scattering assessed the nanoscale size of the formed particles and provided insight in their stability. The nanoparticles’ size was confirmed by direct imaging in transmission electron microscopy, and their crystalline nature was disclosed by X-ray diffraction. The X-ray photoelectron spectroscopy showed measurements compatible with the presence of surface oxide, which was confirmed by the surface potential measurements, which are the novel point of the present work. In conclusion, the method of laser ablation in liquid for the synthesis of gold nanoparticles has been presented, and the advantage of this physical approach, consisting of coating the nanoparticles in situ with gold oxide which provides the required morphological and chemical stability without organic surfactants, has been confirmed by using scanning Kelvin probe microscopy for the first time.

## 1. Introduction

Gold (Au) nanoparticles (NPs) are used in many applications [1,2], from biology-oriented fluorescent labeling for microscopy [3] to biomedical drug-delivery carriers [4] or vectors for thermal cancer treatment [5], on to optoelectronic applications thanks to the plasmonic properties, e.g., for surface-enhanced Raman scattering (SERS) spectroscopy [6,7]. A variety of chemical methods [8,9,10,11,12] have been employed to prepare Au NPs. Nevertheless, the obtained NPs are contaminated with residual by-products such as reducing agents. Pulsed laser ablation (LA) in liquid, a technique born in the 1990s after the seminal work of Henglein and Cotton, among others [13,14], has recently emerged as an alternative approach for the generation of a wide range of inorganic nanomaterials [15,16,17,18,19]. In particular, in our group we focused on the production of high-purity NPs, i.e., without undesired chemicals on their surface [20,21,22,23]. Indeed, the same as for all colloidal NPs, one critical issue is the aggregation and/or instability of Au NPs in suspension, and this is usually addressed by means of organic surfactants [24,25,26]. These organic coatings are effective, yet contaminate the material and may give rise to side effects on the functional properties of the NPs, especially in view of their optical response [27,28] and mechanical or morphological behavior when used, for example, in composites [29,30]. One way to minimize the issues correlated with the organic coating is to provide the metal NPs with an alternative oxide coating soon after synthesis, which is the natural result of the LA technique described in this work [31]. Not only is the oxide passivating layer more stable than any possible organic ligand, due to the intrinsic properties of oxides (i.e., chemical inertness, thermal stability, physical properties such as hardness), but also it is intrinsically limited to the NP surface. In fact, its growth does not require additional passivating material to be inserted into the colloidal solution. As a consequence, there exists no extra coating substance, either suspended in the medium or deposited on the vessel bottom, possibly due to ineffective coating.

In this work, after synthesizing the Au NPs by ultra-fast pulsed LA of an Au target in liquid, we characterized the NPs first in solution, by means of dynamic light scattering (DLS) measurements of both size and zeta-potential, as well as UV-vis spectroscopy. Then, after drop-casting the Au NPs onto solid supporting substrates, the presence of the surface oxide layer was assessed by X-ray photoelectron spectroscopy (XPS), and the NPs’ size was confirmed by atomic force microscopy (AFM). The AFM measurements were further complemented by electrical surface potential measurements of scanning Kelvin probe microscopy (SKPM).

## 2. Results

**Figure 1:**
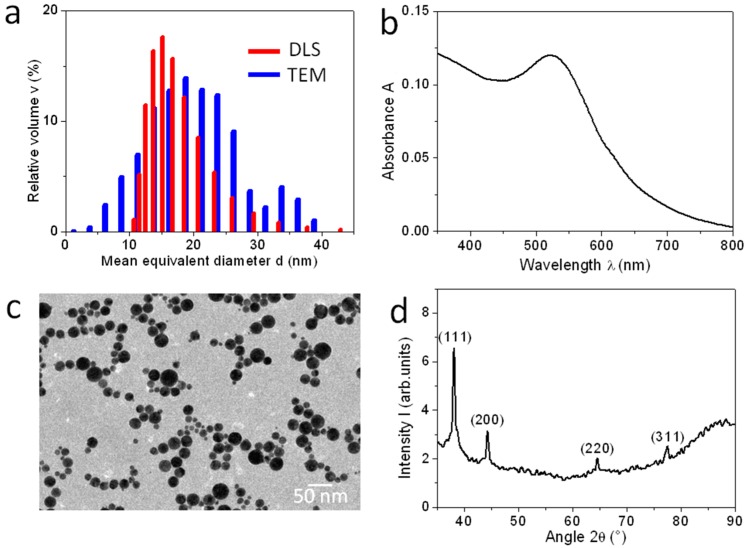
(**a**) Size measurements of the NPs resulting from LA of an Au target, from both DLS of the colloidal solution and analysis of TEM image after drop-casting on silicon substrate; (**b**) UV-vis absorption of the colloidal solution; (**c**) TEM image showing the size of the NPs in the direct space; (**d**) XRD pattern resulting from a sample of drop-cast Au NPs.

In Figure 1, the morphological and structural characterization of the fabricated particles is presented. The particles were first measured in the same liquid medium of the synthesis for assessment of their size by DLS soon after synthesis. The DLS size distribution by volume is presented in red in Figure 1a, and appears to be monomodal with a peak at ~17 nm, covering a diameter range roughly from 10 to 40 nm. The poly-dispersity index was 2.9. While this size population has a spread not as narrow as that obtained from some chemical routes, it still is single since no other peaks around 100 nm or above were observed as in the case of Reference [31], thanks to a relatively inefficient fabrication from the energetic point of view. In fact, in our system we used very different working parameters (namely 1064 nm wavelength, 20 Hz repetition rate, and 60 fs pulse duration) with respect to those used in Reference [31] (800 nm wavelength, 1 kHz repetition rate, 50 fs pulse duration), which are all known to affect the size and size dispersion of the resulting colloidal solution differently. 

The zeta-potential measurement resulted in a value of −33.5 mV, high enough in modulus (≥30 mV) such that an acceptable stability is obtained for the NPs in solution, preventing aggregation thanks to the mutual electrostatic repulsion. The UV-vis spectrum of the colloidal NPs is shown in Figure 1b. An absorbance peak appears at around the 525 nm wavelength, which is typical of the plasmon resonance excited in Au NPs [27,32,33]. The peak also shows no significant broadening and red-shift, which confirms the single size population of the NPs. 

After drying the Au NPs, further characterization was made by transmission electron microscopy (TEM) and X-ray diffraction (XRD). The TEM image in Figure 1c confirms the size distribution of the NPs as described by DLS, mainly in the 10–40 nm range. Indeed, in Figure 1a we also included, in blue, the size distribution resulting from the image analysis of Figure 1c, by volume the same as for the DLS distribution for the best comparison. This distribution largely overlaps with the red one of the DLS, with a peak at slightly a larger value of ~19 nm (and a standard deviation of ~7 nm, probably overestimated due to segmentation issues). In Figure 1d, the XRD spectrum of NPs drop-cast on silicon is presented. The diffraction peaks detected at 38.1°, 44.1° and 64.1° can be indexed to the 111, 200 and 220 planes of the Au structure with a cubic face-centered unit cell. 

**Figure 2:**
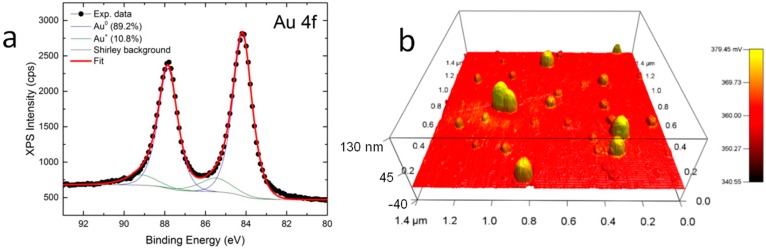
(**a**) XPS measurement of the Au NPs drop-cast onto HOPG, showing the presence of oxidized Au together with metallic Au; (**b**) AFM 3D image of topographic profile of the NPs on silicon, with electrical surface potential overlaid as the color levels.

In Figure 2a the XPS data collected over the energy region typical for Au 4f peaks of a sample of NPs drop-cast on highly oriented pyrolytic graphite (HOPG) are shown. The data should allow for assessing the presence of Au in oxidized form, which corresponds to a different surface state and characteristic binding energy. Indeed, the spectrum is characterized by the presence of two doublets, as obtained by spectral deconvolution of the experimental profile. The most intense one, having the typical 4f_7/2_ and 4f_5/2_ core levels, centered at 84.1 ± 0.2 and 87.8 ± 0.2 eV, respectively, can be assigned to metallic Au, in agreement with the data reported in [27]. 

The best fit to the XPS data has been obtained considering the presence of a second doublet also, accounting for ~11% of the Au content of the surface of the sample. The 4f_7/2_ and 4f_5/2_ peaks of this second doublet are centered at 85.5 ± 0.2 and 89.2 ± 0.2 eV, respectively. The observed positions are in good agreement with the values reported in Reference [31] for the Au^+^ oxidation state and support the partial oxidation of the Au NPs obtained here by LA. 

In Figure 2b a representative AFM/SKPM image of the Au oxide NPs drop-cast on silicon is shown. The three-dimensional (3D) surface represents the topography of the sample (see vertical scale on the left-hand side, in nm). On the atomically flat substrate the NPs clearly appear, and exhibit apparently different sizes, still always within the range of a 50–150 nm diameter. It should be considered here that the NP size in AFM is always laterally enlarged roughly by the tip size, and in particular for SKPM the standard tip diameter (10 nm) is further increased by the metallic coating to typically 60–70 nm. However, we are more interested in the electrical potential image measured simultaneously with the topography in the SKPM mode. On the same image in Figure 2b, the potential map has been used to colorize the 3D profile. As shown by the color bar scale on the right-hand side, yellow represents high potential values as compared to red. It appears that the NPs have co-localized bright potential contrast (values higher than the surrounding silicon). 

## 3. Discussion

**Figure 3:**
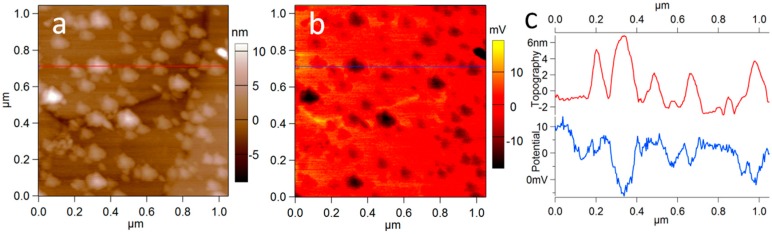
SKPM measurement of commercial (oxide-free) Au NPs on silicon. (**a**) Topography image; (**b**) surface potential image; (**c**) cross-sections at the horizontal red line in (**a**,**b**).

The NPs’ size distributions in Figure 1a, from the DLS scattering model (in red) and from the TEM direct space imaging (in blue), are in good agreement, peaking between 17 and 19 nm. As mentioned previously, the apparent lateral size of NPs in Figure 2b is instead quite larger, 50–150 nm. However, when the AFM image height is considered, which is not affected by the tip width, the NPs’ size range of Figure 2b appears roughly as expected, 10–40 nm. The same holds for the commercial Au NPs (see Figure 3c, red profile), which are considerably smaller (nominal diameter: 5 nm). 

On the other hand, Figure 1b,c are in agreement in assigning the prevailing NP material to Au. Additionally, Au being the NP material is also included as the assumption in DLS modeling, after the material refractive index, and thus all the above measurements in Figure 1 are consistent in assigning the observed particulate matter resulting from our LA synthesis in liquid to Au NPs. 

Concerning the presence of Au oxide on the NP surface, this is supported by the combined XPS and SKPM measurements in Figure 2. Particularly in Figure 2b, the positive potential contrast at the NPs on Au is only possible when they are coated with a dielectric. In a former work, we demonstrated this effect as due to a non-conductive organic surfactant [27]; in the present case, because LA synthesis is carried out in DI water and no surfactant may be present, the observed SKPM effect can only be ascribed to the presence of a surface oxide layer. As visible in the colors of Figure 2b, an effect of the NPs’ size on the surface potential also appears, as the effect of the measured surface potential is additive. Obviously, the above-demonstrated surface oxidation, in agreement with the work by Sylvestre et al. [31], even though partial, is sufficient to prevent excess agglomeration above the 40 nm NPs size in the environmental conditions (solvent, surfactant, temperature) considered here. 

The present SKPM measurement is not quantitative since, even if the SKPM tip was calibrated versus HOPG, a detailed band diagram assignment of the Au-Au oxide system work functions still is not due to the unknown thickness/coverage of the oxide. Nevertheless, the contrast in Figure 2b allows for the qualitative discrimination of the NPs’ coating nature as a dielectric. In fact, in Figure 3a the control measurement of non-oxidized Au NPs from a commercial source is presented. While the NPs in Figure 3 do not appear perfectly round, which can be assigned to a known tip-convolution artifact easily occurring on this small scan size, in this case the Au NPs exhibited negative contrast in the potential image of Figure 2b (see also the respective cross-sections of topography and potential in Figure 3c). This potential contrast at the NPs is opposite to that appearing in Figure 2b for our Au oxide NPs.

Most liquid methods for fabrication of Au NPs are chemical (Turkevic-Frens [8], Brust [9], Perrault [10], Martin [11], Navarro [12]), with only the sonolysis approach being a liquid physical method of top-down nature [34]. LA is a physical liquid method that can be considered a top-down synthesis as well, with respect to the starting bulk metal target. After the ablation explosion plume in the liquid, the strong exothermic process is obviously reconstructing matter removed from the target in the forms of self-organized NPs. Since they do not exhibit organic ligands on their surface, the oxidized Au NPs fabricated by LA are label-free, and thus are optimal for surface-enhanced spectroscopies [35] such as, e.g., SERS, or for similar applications sensitive to contaminants such as in biosensors [36]/immunoassays [37]. For SERS, this point guarantees a featureless Raman background, which is a prerequisite for clear interpretation of the Raman spectra during operation [38]. Indeed, SERS allows sensitivity levels down to single-molecule detection. However, the qualitative detection of the molecular fingerprint may be hindered by the presence or organic contaminants ascribed to the surfactant. 

In conclusion, a detailed characterization of non-chemically synthesized Au NPs has been presented, in particular reporting novel SKPM measurements. While the method of synthesis used in this work operates in liquid medium, it relies on a physical approach based on LA of a bulk solid target. The advantage of LA in liquid consists of providing the Au NPs with a coating of oxide in situ, which has been demonstrated by SKPM. Even if SKPM is not quantitative and cannot be considered as an analytical technique, the presence of Au oxide has been shown to be consistent with XPS measurements. The partial oxide coating imparts the Au NPs the required morphological and chemical stability without the use of organic ligands as surfactants. The same technique of synthesis can be applied to different metals, such as silver or copper. These oxide-stabilized metal NPs may find applications in several areas, from biotechnology to microscopy, on to photonics/optoelectronics, or a combination thereof. 

## 4. Materials and Methods

### 4.1. Au NPs Investigated

Synthesis of ligand-free Au NPs was carried out using a Continuum Leopard laser providing pulses at 1064 nm, with a time duration of 60 ps and at a repetition rate of 20 Hz. The laser beam with a diameter of 6 mm was focused 3 mm below the target surface using a lens with a focal length of 30 cm. Pulse energy was fixed at 10 mJ. The Au target (99.999% from Alpha Aesar, Karlsruhe, Germany), in the form of a cylinder with a diameter of 6 mm and a thickness of 5 mm, was placed on the bottom of a quartz cuvette (dimension 10 × 10 × 30 mm^3^) and immersed in 2 mL of deionized water. The height of the liquid above the target surface was 20 mm. Before experiment the target was mechanically polished and then washed with the same liquid used for the LA several times to remove impurity from the surface. The target was placed on a motorized stage (T-cube DC Servo controller, Thorlabs, Newton, NJ, USA) that moved at a constant speed of 1 mm/s in a spiral with an outer radius of 1 mm. The irradiation time was fixed at 10 min. 

The Au NPs used as a control in Figure 3, for comparison of the resulting SKPM surface potential contrast, are from Sigma-Aldrich (Milan, Italy), product No. 752568. The nominal size was 5 nm, and the reactant-free suspension was stabilized in 0.1 mM phosphate buffer saline. 

### 4.2. NPs Characterization Techniques

#### 4.2.1. In Solution

Optical absorption spectra were recorded in a quartz cuvette (10 mm, Helma, Müllheim, Germany), using a Cary 6000 UV-vis double beam spectrophotometer (Agilent, Cernusco sul Naviglio, Italy).

Hydrodynamic size and zeta-potential measurements of Au-NPs were performed by dynamic light scattering (DLS) with a Zetasizer nano series instrument (Malvern Instruments, Malvern, UK). Soon after synthesis, the colloidal solution of Au NPs was injected into semi-micro disposable quartz cell and into quartz electrophoretic cell, for the direct size and indirect stability evaluation by zeta-potential analysis, respectively. The reported values were obtained after at least three replicate measurements for each sample.

#### 4.2.2. In Dried Form

For the TEM measurements we used a JEM-1011 instrument (JEOL, Tokyo, Japan) equipped with a thermionic electron source (tungsten filament) working at 100 kV. The colloidal NP solution was dropped onto carbon-coated 150-mesh copper-grids CF-150-Cu50 (Electron Microscopy Sciences, Hatfield, PA, USA) and let to dry in ambient air. The grain analysis of the representative TEM image in Figure 1c has been carried out in Igor 6.31 (Wavemetrics, Lake Oswego, OR, USA), setting a minimum object size of 16 pixels (1 √pixel = 0.672 nm) and a threshold of 60 (8 bit image) and approximating the grains to ellipses. 

The XRD measurements were carried out on a X-ray diffractometer SmartLab (Rigaku, Tokyo, Japan) equipped with a 9 kW CuKα (λ = 1.542 Å) rotating anode, working at 40 kV and 150 mA. A Göbel mirror was used to convert the divergent X-ray beam into a parallel beam and to suppress the CuKβ radiation (λ = 1.392 Å). The diffraction patterns were collected at room temperature over an angular range of 5° to 60°, with 0.05° step-size and 2°/min scan speed. 

XPS analyses were carried out with a Kratos Axis Ultra^DLD^ spectrometer using an Al Kα source operated at 20 mA and 15 kV. The Kratos charge neutralizer system was used on all specimens. High resolution analyses were carried out with an analysis area of 300 × 700 microns and pass energy of 10 eV. Spectra have been charge corrected to the main line of the C 1s spectrum (adventitious carbon) set to 284.8 eV. Spectra were analyzed using CasaXPS software (version 2.3.17). Data fitting was performed using Shirley-type background and Gauss-Lorentz profiles. For each Au 4f doublet, a spin-orbit splitting of 3.67 eV and a branching ratio of three-quarters were assumed. 

The SKPM measurements were carried out with an MFP-3D AFM (Asylum Research, Goleta, CA, USA). We used Electrilever AC240TM probes (Olympus, Tokyo, Japan). The cantilevers had typical spring constant and resonance frequency of 2 N/m and 70 kHz, respectively. The tips had nominal length and core diameter of 14 mm and 60 nm, respectively, and were coated with a 30 nm layer of Pt/Ir to provide electrical contact. The measurement was based on the so-called ‘nap’ mode: During a first pass, the cantilever was mechanically driven at its first resonance in air (tapping mode), tracking the surface topography; on the same line, before moving on to the next one, a second pass was carried out, making the tip fly over the sample surface at a constant elevation height Δ*H*. During this ‘nap’ pass the cantilever was driven at the same resonance frequency but by electrical means (applied AC voltage, *V*_AC_). Simultaneously, the tip was also biased with a DC voltage *V*_DC_. The sample was set to ground (*V* = 0). The feedback acted on *V*_DC_ such as to cancel the electrical force F, in such a way that the required voltage to achieve this goal was the surface potential *V*_SP_: *V*_DC_ = −*V*_SP_. The *V*_SP_ measured locally on the sample, in the absence of static charge, is associated with the sample work function ϕ*_sam_*, such that *eV_SP_* = ϕ*_tip_* − ϕ*_sam_*, where *e* is the quantum charge of a single electron and ϕ*_tip_* is the work function of the tip. The latter is a characteristic of the selected tip model, mainly due to the type and thickness of metallic coating. ϕ*_tip_* is measured during a prior calibration step on a featureless (flat) sample with known work function, typically HOPG. For the probes used in this work it was ϕ*_tip_* ≈ 4.6 eV. 

## Abbreviations

The following abbreviations are used in this manuscript:

NPs	nanoparticles
SERS	surface-enhanced Raman scattering
LA	laser ablation
DLS	dynamic light scattering
UV-Vis	ultraviolet-visible
XPS	X-ray photoelectron spectroscopy
AFM	atomic force microscopy
SKPM	scanning Kelvin probe microscopy
